# Malignancy-Associated Pulmonary Embolism: Mortality, Recurrence, and Bleeding Risks

**DOI:** 10.3390/jcm14217819

**Published:** 2025-11-04

**Authors:** Daniela Maria Nemțuț, Florica Voiță-Mekeres, Ruxandra Ulmeanu, Florian Bodog, Grațiela Avram, Ioan Bogdan Voiță, Nuțu Cristian Voiță, Mariana Racoviță, Alexandru Catalin Motofelea, Lavinia Davidescu

**Affiliations:** 1 Doctoral School of Biomedical Sciences, University of Oradea, 410073 Oradea, Romania; danyellaroman@gmail.com (D.M.N.); r_ulmeanu@yahoo.com (R.U.); voita_bogdan@yahoo.com (I.B.V.); voita-cristi@yahoo.com (N.C.V.); 2 Morphological Disciplines, University of Oradea, 410073 Oradea, Romania; 3 Department of Surgical Disciplines, Faculty of Medicine and Pharmacy, University of Oradea, 410073 Oradea, Romania; gavram@uoradea.ro (G.A.); mracovita@uoradea.ro (M.R.); 4 Center for Molecular Research in Nephrology and Vascular Disease, Discipline of Nephrology, Department VII/Internal Medicine II, Faculty of Medicine, “Victor Babes” University of Medicine and Pharmacy, 300041 Timisoara, Romania; alexandru.motofelea@umft.ro; 5 Department of Medical Disciplines, Faculty of Medicine and Pharmacy, University of Oradea, 1 Universitatii Street, 410087 Oradea, Romania; lavinia.davidescu@didactic.uoradea.ro

**Keywords:** pulmonary embolism, direct oral anticoagulants, cancer-associated thrombosis, NT-proBNP

## Abstract

**Background/Objectives**: Pulmonary embolism (PE) remains a leading cause of morbidity and mortality, with outcomes influenced by patient demographics, comorbidities, and anticoagulation strategy. While vitamin K antagonists (VKA) have been standard therapy, direct oral anticoagulants (DOACs) are increasingly adopted, yet real-world data in cancer-associated and non-cancer populations are limited. This study aimed to compare demographics, clinical features, therapeutic strategies, and outcomes between oncologic patients with acute PE (experimental group) and non-cancer patients with PE (control group). **Methods**: We performed a multicentric retrospective cohort study of adults admitted with acute PE between January 2019 and December 2021. The cohort comprised 120 non-cancer and 106 cancer patients. Standard management was low-molecular-weight heparin with transition to (VKA) or (DOAC), when not contraindicated. Data on demographics, comorbidities, and laboratory biomarkers (including NT-proBNP, threshold 600 pg/mL) were analyzed. Primary outcomes were early (≤30 days) and late (31–365 days) all-cause mortality. Secondary outcomes included PE recurrence and bleeding events. **Results**: Among 226 PE patients (non-oncological n = 120; oncological n = 106), the cancer group was older (69.2 ± 12.6 vs. 62.6 ± 17.3 years; *p* = 0.001) with similar ECG findings and hemodynamic stability at presentation. NT-proBNP > 600 pg/mL was more frequent in cancer (37.7% vs. 23.3%; *p* = 0.018), whereas D-dimer > 5 mg/L was more common in non-cancer (74.2% vs. 55.7%; *p* = 0.003). DOAC use was lower in cancer patients (40.6% vs. 65.0%; *p* < 0.001). Early mortality was comparable (17.9% vs. 13.3%; *p* = 0.341), but late mortality was higher in the cancer patient subgroup (38.7% vs. 3.3%; *p* < 0.001). In multivariable analysis, belonging to the cancer subgroup was associated with NT-proBNP ≥ 600 (OR 2.08, 95% CI 1.08–4.01; *p* = 0.029) and older age (OR 1.025 per year, 95% CI 1.005–1.045; *p* = 0.016), and inversely associated with D-dimer > 5 mg/L (OR 0.35, 95% CI 0.19–0.64; *p* < 0.001). **Conclusions**: Prompt anticoagulation was associated with lower early mortality, while differences in late mortality appeared to be largely confounded by age and cancer status. NT-proBNP may serve as a useful risk-stratification biomarker, but confirmation in larger, prospective studies is required.

## 1. Introduction

Every year, approximately 100,000 people in the US lose their lives to pulmonary embolisms (PE), a potentially fatal condition [1]. According to recent data, the mortality rate for PE rose from 2008 to 2018 with 0.6% per year, a trend thought to reflect both greater disease recognition and improved diagnostic sensitivity rather than a true worsening of case fatality [2].

Disparities have also been found among various demographic groups, which could influence PE treatment approaches and results [2,3]. Anticoagulation and thrombolysis are advised for patients with extensive/limb-threatening deep vein thrombosis (DVT) or PE accompanied by high-risk PE [4,5].

This high-risk PE is defined as systemic hypotension (systolic arterial pressure < 90 mmHg or a decrease of at least 40 mmHg for ≥15 min not caused by new arrhythmias or shock) [6]. It is not clear whether right ventricular dysfunction or cardiac necrosis and thrombolysis are present in this patient group, but it is generally considered on a case-by-case basis [7]. Thrombolysis may be administered either systemically through an intravenous (IV) infusion, usually preferred in high-risk PE patients because it is readily available, or via catheter-directed delivery, which allows local infusion of fibrinolytics at lower doses with a safer profile [4].

In patients with active malignancies, however, the decision to use thrombolysis is far more complex. Although it can be life-saving in massive PE, these patients face a markedly higher risk of serious bleeding events, including intracranial hemorrhage, because of tumor-related vascular fragility, recent surgeries, metastases, and treatment-induced thrombocytopenia [8,9]. For this reason, systemic thrombolysis is often avoided unless the situation is truly life-threatening, and when considered, the potential benefits must be weighed very carefully against the risks. Whenever possible, clinicians tend to favor catheter-directed approaches or alternative strategies that may offer a safer balance [10].

Anticoagulation therapy significantly reduces the risk of recurrent venous thromboembolism (VTE) by 80% to 90% [7], yet it is associated with a 1% to 3% annual risk of major bleeding (MB) [11,12]. The most severe forms of anticoagulation-related bleeding include fatal and intracranial hemorrhages, which constitute 13.4% and 8.7% of MBs, respectively [12]. Additionally, anticoagulation-related MB is linked to considerable healthcare costs and a diminished quality of life [13,14,15]. Previous research has indicated that the risk of anticoagulation-related bleeding is not constant, with a heightened risk of MB during the initial phase compared to later periods of anticoagulation [12,16]. Observational studies have documented the incidence and risk factors for bleeding in specific populations, such as individuals with atrial fibrillation, elderly patients with renal impairment, or those on particular anticoagulants [17]. Recent comprehensive studies have highlighted the safety profiles of various direct oral anticoagulants (DOACs) in patients diagnosed with atrial fibrillation. Rather than emphasizing one specific agent, accumulating evidence suggests that DOACs as a class are associated with lower rates of major bleeding compared to vitamin K antagonists (VKA), although head-to-head differences between DOACs remain debated [18,19,20]. Underlining the need for clarity in real-world practice, most of the available data on anticoagulation safety come from randomized trials or selected registries, while less is known about unselected hospital cohorts or patients with concurrent cancer.

Further evidence for the preferential use of apixaban in high-risk populations comes from a European registry analysis, which emphasized its superior bleeding profile among the DOACs [18,21]. These findings are consistent with contemporary literature that endorses apixaban’s safety advantages over rivaroxaban and dabigatran [18,19]. Additionally, real-world data, such as studies indicating that apixaban is associated with reduced major bleeding risks compared to other anticoagulants, strengthen the case for its preferred use [20,22]. The pharmacokinetic profile of apixaban, including fixed dosing and its twice-daily administration, may also contribute to its efficacy in minimizing bleeding risks compared to rivaroxaban, which is administered once daily and may exhibit greater peak-trough fluctuations in plasma levels [21,22].

This study aimed to compare demographics, comorbidities, diagnostic biomarkers (ECG, NT-proBNP, D-dimer), anticoagulation strategies, and outcomes (early/late mortality, recurrence, major bleeding) in oncological versus non-oncological patients with acute PE, stratified by treatment with LMWH followed by VKA or DOACs.

## 2. Materials and Methods

### 2.1. Study Design and Ethics

This multicentric, retrospective cohort study analyzed adult patients admitted with acute PE between 1 January 2019, and December 31, 2021. The study protocol was approved by the Ethics Committee of the Oradea County Emergency Clinical Hospital (registration number 30278/12.12.2019), the Ethics Committee of the Municipal Hospital “Dr. Gavril Curteanu” Oradea (registration number 31465/05.11.2020). Written informed consent was obtained from all patients.

### 2.2. Study Population

Patients aged 18 years or older with a primary diagnosis of PE confirmed by computed tomography pulmonary angiography (CTPA) were identified through electronic medical records. The final sample included 226 patients, divided into two groups. The cohort included 120 non-cancer and 106 cancer patients. Management typically involved LMWH with planned transition to VKA or DOAC; in selected cases, anticoagulation was withheld or delayed due to contraindications or early deterioration.

### 2.3. Inclusion and Exclusion Criteria

Inclusion criteria comprised patients with acute PE with or without associated oncological pathology, regardless of tumor location, stage, or treatment modality including surgery, chemotherapy, or radiotherapy. Patients with recurrent PE were also included under the same conditions. Patients over 18 years of age with acute PE were eligible: those without significant comorbidities were assigned to the control group, and those with oncologic pathology to the oncological group. Exclusion criteria encompassed patients without PE irrespective of oncological status, refusal to participate, presence of psychiatric pathology interfering with participation, and age under 18 years.

### 2.4. Data Collection

Baseline demographic information included age, sex, and residency status categorized as rural or urban based on patient address. Clinical variables incorporated comorbidities such as hypertension, diabetes mellitus, obesity defined by a body mass index of 30 kg/m^2^ or greater, pulmonary diseases including chronic obstructive pulmonary disease or interstitial lung disease, cardiovascular disease defined by history of coronary artery disease or heart failure, neurological disorders including stroke or neurodegenerative diseases, renal impairment as estimated glomerular filtration rate below 30 mL/min/1.73 m^2^, and gastrointestinal comorbidities characterized by active inflammatory or neoplastic conditions documented at admission. Embolic site was classified as lobar, main pulmonary artery, segmental, or combined involvement based on radiology reports. Diagnostic investigations included electrocardiography, measurement of proBNP levels, and D-dimer assays performed at presentation. NT-proBNP levels were measured at admission as a marker of right ventricular strain and hemodynamic stress, both of which are prognostically relevant in acute PE. A threshold of 600 pg/mL was applied, consistent with previous studies and guideline-based evidence, where values above this level have been associated with higher risk of adverse outcomes [23,24].

Hemodynamic instability was defined as systolic blood pressure less than 90 mmHg or a decrease of 40 mmHg or more lasting at least 15 min without arrhythmia as cause [25]. Unstable clinical status was further characterized by low cardiac output syndrome, arterial hypotension, cardiogenic shock with or without hypoxemia, or cardio-respiratory arrest. Although we did not apply a formal ESC risk class stratification, patients were categorized according to hemodynamic status and biomarker profile (NT-proBNP levels), which are established prognostic indicators in acute PE. This approach allowed us to identify high-risk and unstable patients within our cohort.

Electrocardiographic (ECG) changes analyzed included sinus tachycardia, right axis deviation, complete or incomplete right bundle branch block, the presence of Q waves, negative T waves in leads DIII and V1 through V4, P pulmonale, and the S1Q3T3 pattern.

The primary endpoint was early death, defined as all-cause mortality within 30 days of PE diagnosis [6]. Late mortality was defined as death 31–365 days after diagnosis, consistent with contemporary PE outcomes literature [26]. Secondary outcomes included rates of thrombolytic therapy and clinical recurrence of PE within one year.

### 2.5. Statistical Analysis

Continuous variables are presented as mean values with standard deviations and compared using Student’s *t*-test. Categorical variables are reported as counts and percentages and compared using the chi-square test or Fisher’s exact test as appropriate. The Shapiro–Wilk test was conducted to assess the sample distribution. To identify independent factors associated with cancer status (oncological vs. non-oncological) among patients with acute PE, we fit a multivariable logistic regression model using maximum-likelihood estimation. Covariates measured at presentation included age (continuous) and the following categorical variables: NT-proBNP (≥600 vs. <600 pg/mL), D-dimer (>5 mg/L FEU vs. ≤5 mg/L FEU), sex, ECG changes (yes/no), hemodynamic status (stable/unstable), environment (urban/rural), and pulmonary hypertension (yes/no). Model adequacy was assessed using residual deviance, Akaike Information Criterion (AIC), and McFadden’s pseudo-R^2^. Results are reported as adjusted odds ratios (ORs) with 95% confidence intervals (CIs), alongside the corresponding regression coefficients (β), standard errors (SE), Wald *z* statistics, and two-sided *p* values. A two-tailed *p*-value of less than 0.05 was considered statistically significant. All analyses were performed using R version 4.1.2.

## 3. Results

The cohort comprised 226 patients, assigned to two groups: non-oncological patients (n = 120) and oncological patients (n = 106). The oncological group was older (mean 69.2 ± 12.6 years) than the non-oncological group (62.6 ± 17.3 years; *p* = 0.001); the overall mean age was 65.7 ± 15.6 years. Gender distribution did not differ significantly (female: 54.7% vs. 44.2%; *p* = 0.113).

Residence was similar between groups: 46 oncological patients (43.4%) versus 58 non-oncological patients (48.3%) resided in rural areas (*p* = 0.457) (Table 1).

In the cancer subgroup (n = 106), the most common primary sites were digestive 31/106 (29.2%), lung 28/106 (26.4%), and urogenital 26/106 (24.5%), with smaller contributions from breast 8/106 (7.5%), lymphomas 6/106 (5.7%), and other sites 7/106 (6.6%). Tumor stage was unavailable for 27/106 (25.5%). Among the 79 staged cases, disease was largely advanced—46/79 (58.2%) were T4 and 21/79 (26.6%) were T3, i.e., 67/79 (84.8%) T3–T4 overall, which corresponds to 67/106 (63.2%) of the entire cancer cohort. PE occurred during high-risk windows: at initial cancer diagnosis in 38/106 (35.8%), after chemotherapy in 37/106 (34.9%), and after surgery in 29/106 (27.4%) (these categories are not mutually exclusive). Only 2/106 (1.9%) were receiving hormonotherapy and 8/106 (7.5%) had radiotherapy recorded around the PE event. Collectively, the predominance of digestive/lung primaries, the high proportion of advanced-stage disease, and the clustering of PE around diagnosis, chemotherapy, and surgery plausibly explain the elevated thrombotic risk and higher late mortality in this group; the 27 unstaged cases limit finer risk stratification by tumor burden.

ECG findings revealed no significant differences between groups, with no abnormalities observed in 76 non-oncological patients (63.3%) compared with 57 oncological patients (53.8%) (*p* = 0.145). Changes specific to PE were present in 44 non-oncological patients (36.7%) and 49 oncological patients (46.2%), representing 41.2% of the total co-hort. NT-proBNP levels differed significantly, with values > 600 pg/mL in 40 oncological patients (37.7%) compared with 28 non-oncological patients (23.3%) (*p* = 0.018). Conversely, levels < 600 pg/mL were more frequent in the non-oncological group (92 patients, 76.7%) than in the oncological group (66 patients, 62.3%).

Hemodynamic status was evenly distributed between groups. Unstable status was recorded in 25 non-oncological patients (20.8%) and 22 oncological patients (20.8%) (*p* = 0.988). Most patients were hemodynamically stable: 95 (79.2%) in the non-oncological group and 84 (79.2%) in the oncological group.

The prevalence of pulmonary hypertension was similar across cohorts, affecting 93 non-oncological patients (77.5%) and 81 oncological patients (76.4%) (*p* = 0.847).

D-dimer levels > 5 mg/L were significantly more common in the non-oncological group, observed in 89 patients (74.2%) compared with 59 patients (55.7%) in the oncological group (*p* = 0.003). Conversely, D-dimer levels < 5 m/mL were more frequent in the oncological group (47 patients, 44.3%) than in the non-oncological group (31 patients, 25.8%) (Table 2).

Hypertension was more frequent in the oncological group than in the non-oncological group (50.9% vs. 39.2%), although the difference did not reach statistical significance (*p* = 0.076). Diabetes mellitus occurred in 17.0% and 13.3% of patients, respectively (*p* = 0.444).

Obesity showed a significant between-group difference, being more common in the non-oncological group (19.2% vs. 5.7%; *p* = 0.002). No significant variation was observed for cardiovascular disease (37.7% vs. 40.8%; *p* = 0.634), neurological disorders (9.4% vs. 9.2%; *p* = 0.945), or pulmonary disease (21.7% vs. 16.7%; *p* = 0.336).

Peripheral vascular disorders were less frequent in the oncological group (20.8% vs. 35.0%; *p* = 0.018). Gastrointestinal, renal, and other comorbidities were uncommon and showed no significant differences between groups (*p* = 0.187, 0.450, and 0.182, respectively) (Table 3).

Lobar embolism was the most frequent localization, occurring in 67.0% of the oncological group and 52.5% of the non-oncological group, a difference that approached but did not reach statistical significance (*p* = 0.081). Main pulmonary artery involvement was observed in 31.7% and 22.6% of patients, respectively, while combined main and lobar emboli were rare (2.5% vs. 0%). Segmental embolism was the least common site (13.3% vs. 10.4%).

Anticoagulant use differed significantly between groups. A greater proportion of oncological patients were not on DOAC therapy (59.4% vs. 35.0%; *p* < 0.001). Among specific agents, no significant differences were observed for Acenocoumarol (22.5% vs. 27.4%; *p* = 0.399) or Apixaban (25.0% vs. 27.4%; *p* = 0.687). By contrast, Rivaroxaban (20.8% vs. 8.5%; *p* = 0.010) and Dabigatran (19.2% vs. 4.7%; *p* = 0.001) were more frequently prescribed in the non-oncological group. LMWH use was comparable between groups (35.0% vs. 32.1%; *p* = 0.642) (Table 4).

Early mortality (≤30 days) was similar between groups: 17.9% in oncological vs. 13.3% in non-oncological patients (*p* = 0.341). Late mortality (31–365 days) was substantially higher in oncological patients—38.7% vs. 3.3% in non-oncological patients (*p* < 0.001). Thrombolysis was uncommon overall and numerically less frequent in oncological patients (1.9% vs. 5.8%; *p* = 0.130). Recurrent PE at 1 year was infrequent and not significantly different: 7.5% in oncological vs. 3.3% in non-oncological patients (*p* = 0.159) (Table 5).

Table 6 shows that anticoagulation was strongly associated with lower early mortality in both cohorts. Among non-oncological patients, early death occurred in 33.3% of those not anticoagulated versus 2.6% of those anticoagulated (*p* < 0.001); among oncological patients, the corresponding rates were 28.6% and 2.3% (*p* < 0.001). By contrast, late mortality did not differ by anticoagulation status within either cohort: 2.4% versus 3.8% in non-oncological patients (*p* = 0.670) and 41.3% versus 34.9% in oncological patients (*p* = 0.507). Notably, absolute late mortality remained substantially higher in the oncological group overall, consistent with cancer-related risk. Recurrent PE was infrequent and showed no significant association with anticoagulation status—4.8% versus 2.6% in non-oncological patients (*p* = 0.522) and 9.5% versus 4.7% in oncological patients (*p* = 0.351). Bleeding events were numerically more common when anticoagulation was withheld in both cohorts (non-oncological: 9.5% vs. 2.6%, *p* = 0.095; oncological: 15.9% vs. 7.0%, *p* = 0.170), although these differences did not reach statistical significance and may reflect confounding by indication.

The association between NT-proBNP levels and clinical outcomes was assessed (Table 7). In the oncological group, early mortality did not differ by NT-proBNP status (18.2% vs. 17.5%; *p* = 0.929). In the non-oncological group, however, early mortality was significantly higher in patients with NT-proBNP > 600 pg/mL compared with those below this threshold (28.6% vs. 8.7%; *p* = 0.007), indicating a prognostic role limited to the non-oncological cohort.

Late mortality showed no significant association with NT-proBNP levels in either group. Among oncological patients, rates were 39.4% versus 37.5% (*p* = 0.846), while in non-oncological patients, late mortality was rare (4.3% vs. 0%; *p* = 0.262).

Recurrence of PE was likewise unaffected by NT-proBNP status. In the oncological group, recurrence occurred in 10.6% versus 2.5% (*p* = 0.126), and in the non-oncological group in 4.3% versus 0% (*p* = 0.262).

Bleeding events were more frequent in patients with elevated NT-proBNP in both groups, though the association reached significance only in the non-oncological cohort. Rates were 9.1% versus 17.5% in the oncological group (*p* = 0.201), and 14.3% versus 2.2% in the non-oncological group (*p* = 0.010).

In multivariable analysis within the PE cohort, NT-proBNP ≥ 600 pg/mL was associated with higher odds of belonging to the cancer subgroup (OR 2.08, 95% CI 1.08–4.01; *p* = 0.029), and age increased the odds by ~2.5% per year (OR per year 1.03, 95% CI 1.01–1.05; *p* = 0.016), whereas D-dimer > 5 mg/L (FEU) was inversely associated (OR 0.35, 95% CI 0.19–0.64; *p* < 0.001). Gender, ECG changes, hemodynamic status, pulmonary hypertension, and environment were not independently associated (Table 8).

## 4. Discussion

This single-center retrospective cohort study compared oncological and non-oncological adults with acute PE and found broadly similar early (≤30 days) all-cause mortality, but a striking divergence in late (31–365 days) mortality. Early mortality did not differ significantly by cancer status (oncological 17.9% vs. non-oncological 13.3%; *p* = 0.341), whereas late mortality was markedly higher in the oncological cohort (38.7% vs. 3.3%; *p* < 0.001). These differences likely reflect baseline risk rather than treatment effects: factors such as older age, comorbidity burden, and cancer-related complications can influence outcomes and confound direct comparisons between anticoagulation strategies [24].

The comparable early mortality suggests that initial PE severity and short-term management can achieve similar outcomes in patients with and without cancer when anticoagulation is administered. The excess late mortality in cancer patients likely reflects underlying malignancy, cancer treatment, related complications, and competing risks rather than PE-specific factors consistent with the lack of difference in 1-year PE recurrence between groups [27,28,29].

The strong reduction in early death among anticoagulated patients in both cohorts underscores that timely anticoagulation is the dominant driver of short-term survival. The numerically higher bleeding when anticoagulation was withheld likely represents confounding by indication rather than a protective effect of non-treatment [30,31,32].

Patients with cancer were less often treated with DOACs and more frequently received non-DOAC regimens, aligning with real-world caution about drug–drug interactions, mucosal bleeding risk (e.g., GI/GU), and historical preference for LMWH in malignancy-associated VTE. In this dataset, agent-specific differences were not powered to show outcome separation. The similar LMWH use across groups and the lower use of rivaroxaban/dabigatran in cancer suggest clinician selection patterns rather than intrinsic differences in presentation severity [27,32,33].

Two biomarker signals diverged by cancer status. First, NT-proBNP > 600 pg/mL predicted early mortality only in non-oncological patients. In cancer, the relationship may be diluted by competing causes of death, chronic elevations from anemia/chemotherapy-related cardiotoxicity, or limited right-ventricular strain discrimination at a single cutoff. Second, D-dimer > 5 mg/L was more frequent in non-oncological patients, which is counterintuitive given cancer-associated coagulopathy. This may reflect spectrum bias (e.g., greater use of imaging at lower D-dimer thresholds in cancer), timing of sampling relative to anticoagulation initiation, or differences in clot burden not fully captured by the categorical localization data [34,35,36].

Lobar involvement trended higher in cancer but without statistical significance, and ECG changes compatible with PE were similar. Together with comparable hemodynamic stability at presentation, these findings reinforce that initial clinical severity was not substantially different by cancer status in this cohort [36,37].

Because CTPA confirmation was uniform, between-group contrasts reflect baseline diagnostic marker profiles (ECG, NT-proBNP, D-dimer) rather than differences in diagnostic pathways. The group underwent D-dimer testing more frequently but exhibited lower rates of ECG patterns and a higher incidence of hemodynamic instability at presentation.

Baseline imbalances likely shaped the mortality patterns observed, introducing bias and limiting causal interpretation of treatment effects. Without multivariable adjustment, differences in age, comorbidities, and malignancy may account for much of the apparent disparity in survival.

The observed strong association between absence of anticoagulation and increased early mortality in both cohorts underscores the vital importance of timely and appropriate anticoagulant therapy in managing PE. Importantly, bleeding rates did not differ significantly according to anticoagulation status, supporting the relative safety of both VKAs and DOAC when used judiciously in selected patients. Because our sample size limited meaningful comparisons between individual DOACs, we chose not to overinterpret apparent differences between apixaban, rivaroxaban, or dabigatran; instead, our results reinforce the need for larger, real-world studies to clarify subtype-specific safety profiles.

Our results are broadly consistent with randomized trials demonstrating the efficacy and safety of DOACs in acute PE, yet they also highlight the high late mortality seen in cancer-associated PE, which is unlikely to be treatment-related. In addition, geographic and socioeconomic differences may influence outcomes and deserve further study. Although cancer status was not stratified in all analyses, exploratory data suggest it may have contributed to the higher late mortality observed in DOAC-treated patients—an area for future dedicated investigation.

NT-proBNP can be regarded as a reliable indicator of short-term mortality risk. Further studies are needed to establish its role in guiding thrombolysis decisions and identifying patients suitable for outpatient treatment [25,26]. Prior studies and meta-analyses show that elevated natriuretic peptide levels are consistently associated with poorer outcomes, and the 600 pg/mL threshold is widely applied in risk stratification [28]. A threshold of 600 ng/L is widely used to stratify risk in hemodynamically stable patients, with levels above this threshold associated with right ventricular dysfunction and higher mortality, while lower levels predict favorable outcomes [29,30]. Our observations align with prior cancer-specific work showing that NT-proBNP ≥ 600 pg/mL marks increased PE recurrence risk, particularly with main pulmonary artery involvement and hemodynamic instability [38]. In our cohort, recurrence was higher in the same direction (10.6% vs. 2.5%) but did not reach statistical significance, likely due to limited events, supporting the need for prospective confirmation. NT-proBNP was predictive of early death and bleeding in non-oncological patients but not in those with cancer, suggesting that its prognostic value may depend on underlying pathology and competing risks. Taken together, these data support NT-proBNP as a simple, inexpensive biomarker to help identify higher-risk patients in routine practice. This finding aligns with existing literature that identifies NT-proBNP as a marker of cardiac strain and adverse prognosis in PE, particularly among patients without complex oncologic comorbidities [25,26,27].

Notably, patients with elevated NT-proBNP, right ventricular dysfunction, and higher PESI scores may benefit from early thrombolytic therapy [30]. Conversely, low NT-proBNP levels can help identify patients suitable for outpatient management. Prior studies also demonstrate a strong association between elevated NT-proBNP and right ventricular dysfunction, which our findings reinforce [26,39,40,41]. Specifically, higher NT-proBNP levels (>600 ng/L) were linked to increased hemodynamic instability and poorer prognosis, indicating a greater risk for both short-term and long-term complications [31].

Overall, NT-proBNP should be regarded as a valuable prognostic tool in acute PE. Despite its utility, its predictive accuracy for long-term outcomes in cancer patients remains limited. Given its accessibility and low cost, routine measurement at diagnosis may improve risk stratification, reduce complications, and support individualized management—though further prospective validation in cancer-associated PE is required [42,43].

## 5. Limitation of the Study

Another limitation is that our initial aims included an evaluation of diagnostic strategies and cancer-specific outcomes, but the available data permitted only partial exploration of these domains. We therefore interpret our findings as hypothesis-generating and encourage future prospective studies with stratified designs to address these gaps. In addition, age distribution and cancer prevalence differed between groups; although both factors are well-established prognostic determinants, our analysis did not include a full multivariable adjustment. As a result, the mortality comparisons should be interpreted with caution. Finally, detailed oncologic information—such as tumor type, stage, and treatment history—was not available, despite its clear influence on outcomes in cancer-associated PE. This missing information further limits interpretation of the higher late mortality observed in the DOAC cohort.

Strengths of this study include the detailed baseline characterization, comprehensive outcome capture, and its relevance to real-world clinical practice. However, limitations such as the retrospective design, single-center setting, and potential unmeasured confounders—including socioeconomic status and imaging data—restrict causal inference and generalizability.

## 6. Conclusions

This study underscores the profound impact of demographic and clinical disparities on PE outcomes, revealing that patient age, geographic background, and treatment modality critically shape survival trajectories. Our findings demonstrate that while traditional VKA-based anticoagulation remains relevant, the growing adoption of DOAC particularly in younger, urban populations offers promising short-term benefits but may be associated with increased late mortality, especially in cancer-associated PE. Our findings suggest that NT-proBNP may serve as a useful prognostic biomarker in acute PE, but further validation is needed in larger, prospective cohorts.

Our observations point to a possible role for patient demographics, comorbidities, and biomarkers in guiding treatment decisions, a question that future research should examine further. Moreover, the marked rural-urban disparities call for healthcare policy interventions aimed at ensuring equitable access to advanced diagnostics and therapies.

Future prospective, randomized studies with rigorous stratification and biomarker integration are imperative to unravel the complex interplay of treatment effects and patient characteristics. Future prospective studies with larger and more diverse populations are needed to confirm these findings and to clarify how treatment strategies and biomarkers can be integrated to improve patient care.

## Figures and Tables

**Table 1:** Demographic and Clinical Characteristics by Cancer Status.

Variable	Category	Non-Oncological Patients (N = 120)	Oncological Patients (N = 106)	Total (N = 226)	*p*-Value
Age (years)	Mean (SD)	62.6 (17.3)	69.2 (12.6)	65.7 (15.6)	0.001
Age Group	<50 years	32 (26.7%)	8 (7.5%)	40 (17.7%)	0.004
	51–60 years	14 (11.7%)	11 (10.4%)	25 (11.1%)	
	61–70 years	26 (21.7%)	35 (33.0%)	61 (27.0%)	
	71–80 years	31 (25.8%)	35 (33.0%)	66 (29.2%)	
	>80 years	17 (14.2%)	17 (16.0%)	34 (15.0%)	
Gender	Female	53 (44.2%)	58 (54.7%)	111 (49.1%)	0.113
	Male	67 (55.8%)	48 (45.3%)	115 (50.9%)	
Environment	Rural	58 (48.3%)	46 (43.4%)	104 (46.0%)	0.457
	Urban	62 (51.7%)	60 (56.6%)	122 (54.0%)	

Pearson’s Chi-squared test.

**Table 2:** Comparison of Electrocardiographic Findings, Biomarkers, Hemodynamic Status, and Pulmonary Hypertension by Cancer Status.

Variable	Category	Non-oncological Patients (N = 120)	Oncological Patients (N = 106)	Total (N = 226)	*p*-Value
ECG Findings	No abnormalities	76 (63.3%)	57 (53.8%)	133 (58.8%)	0.145
	Changes specific to pulmonary embolism	44 (36.7%)	49 (46.2%)	93 (41.2%)	
NT-proBNP	<600 pg/mL	92 (76.7%)	66 (62.3%)	158 (69.9%)	0.018
	>600 pg/mL	28 (23.3%)	40 (37.7%)	68 (30.1%)	
Hemodynamic Status	Unstable	25 (20.8%)	22 (20.8%)	47 (20.8%)	0.988
	Stable	95 (79.2%)	84 (79.2%)	179 (79.2%)	
Pulmonary Hypertension (HTP)	No	27 (22.5%)	25 (23.6%)	52 (23.0%)	0.847
	Yes	93 (77.5%)	81 (76.4%)	174 (77.0%)	
D-dimer	<5 mg/L	31 (25.8%)	47 (44.3%)	78 (34.5%)	0.003
	>5 mg/L	89 (74.2%)	59 (55.7%)	148 (65.5%)	

**Table 3:** Comorbidities distributed by Cancer Status.

Variable	Category	Non-Oncological Patients (N = 120)	Oncological Patients (N = 106)	Total (N = 226)	*p*-Value
Hypertension (HTN)	No	73 (60.8%)	52 (49.1%)	125 (55.3%)	0.076
	Yes	47 (39.2%)	54 (50.9%)	101 (44.7%)	
Diabetes Mellitus (DM)	No	104 (86.7%)	88 (83.0%)	192 (85.0%)	0.444
	Yes	16 (13.3%)	18 (17.0%)	34 (15.0%)	
Obesity	No	97 (80.8%)	100 (94.3%)	197 (87.2%)	0.002
	Yes	23 (19.2%)	6 (5.7%)	29 (12.8%)	
Cardiovascular Disease	No	71 (59.2%)	66 (62.3%)	137 (60.6%)	0.634
	Yes	49 (40.8%)	40 (37.7%)	89 (39.4%)	
Neurological Disorders	No	109 (90.8%)	96 (90.6%)	205 (90.7%)	0.945
	Yes	11 (9.2%)	10 (9.4%)	21 (9.3%)	
Pulmonary Disease	No	100 (83.3%)	83 (78.3%)	183 (81.0%)	0.336
	Yes	20 (16.7%)	23 (21.7%)	43 (19.0%)	
Peripheral vascular disorders	No	78 (65.0%)	84 (79.2%)	162 (71.7%)	0.018
	Yes	42 (35.0%)	22 (20.8%)	64 (28.3%)	
Gastrointestinal Disorders	No	118 (98.3%)	101 (95.3%)	219 (96.9%)	0.187
	Yes	2 (1.7%)	5 (4.7%)	7 (3.1%)	
Renal Disease	No	107 (89.2%)	91 (85.8%)	198 (87.6%)	0.450
	Yes	13 (10.8%)	15 (14.2%)	28 (12.4%)	
Other Comorbidities	No	118 (98.3%)	106 (100.0%)	224 (99.1%)	0.182
	Yes	2 (1.7%)	0 (0.0%)	2 (0.9%)	

Pearson’s Chi-squared test.

**Table 4:** Anticoagulation Therapy by Cancer Status.

Variable	Category	Non-Oncological Patients (N = 120)	Oncological Patients (N = 106)	Total (N = 226)	*p*-Value
Novel Oral Anticoagulants	No	42 (35.0%)	63 (59.4%)	105 (46.5%)	<0.001
	Yes	78 (65.0%)	43 (40.6%)	121 (53.5%)	
Acenocoumarol	No	93 (77.5%)	77 (72.6%)	170 (75.2%)	0.399
	Yes	27 (22.5%)	29 (27.4%)	56 (24.8%)	
Apixaban	No	90 (75.0%)	77 (72.6%)	167 (73.9%)	0.687
	Yes	30 (25.0%)	29 (27.4%)	59 (26.1%)	
Rivaroxaban	No	95 (79.2%)	97 (91.5%)	192 (85.0%)	0.010
	Yes	25 (20.8%)	9 (8.5%)	34 (15.0%)	
Dabigatran	No	97 (80.8%)	101 (95.3%)	198 (87.6%)	0.001
	Yes	23 (19.2%)	5 (4.7%)	28 (12.4%)	
Low-Molecular-Weight Heparin	No	78 (65.0%)	72 (67.9%)	150 (66.4%)	0.642
	Yes	42 (35.0%)	34 (32.1%)	76 (33.6%)	

**Table 5:** Outcomes: Early Death, Late Death, Thrombolysis, Recurrence.

Variable	Category	Non-Oncological Patients (N = 120)	Oncological Patients (N = 106)	Total (N = 226)	*p*-Value
Early Death	No	104 (86.7%)	87 (82.1%)	191 (84.5%)	0.341
	Yes	16 (13.3%)	19 (17.9%)	35 (15.5%)	
Late Death	No	116 (96.7%)	65 (61.3%)	181 (80.1%)	<0.001
	Yes	4 (3.3%)	41 (38.7%)	45 (19.9%)	
Thrombolysis	No	113 (94.2%)	104 (98.1%)	217 (96.0%)	0.130
	Yes	7 (5.8%)	2 (1.9%)	9 (4.0%)	
Recurrence	No	116 (96.7%)	98 (92.5%)	214 (94.7%)	0.159
	Yes	4 (3.3%)	8 (7.5%)	12 (5.3%)	

**Table 6:** Clinical outcomes stratified by oncological vs. non-oncological patients with acute PE.

Outcome	Category	Non-Oncological Patients (No, N = 42)	Non-Oncological Patients (Yes, N = 78)	Non-Oncological Patients Total (N = 120)	*p*-Value	Oncological Patients (No, N = 63)	Oncological Patients (Yes, N = 43)	Oncological Patients Total (N = 106)	*p*-Value
Early Death	No	28 (66.7%)	76 (97.4%)	104 (86.7%)	<0.001 ^1^	45 (71.4%)	42 (97.7%)	87 (82.1%)	<0.001 ^1^
	Yes	14 (33.3%)	2 (2.6%)	16 (13.3%)		18 (28.6%)	1 (2.3%)	19 (17.9%)	
Late Death	No	41 (97.6%)	75 (96.2%)	116 (96.7%)	0.670 ^1^	37 (58.7%)	28 (65.1%)	65 (61.3%)	0.507 ^1^
	Yes	1 (2.4%)	3 (3.8%)	4 (3.3%)		26 (41.3%)	15 (34.9%)	41 (38.7%)	
Recurrence	No	40 (95.2%)	76 (97.4%)	116 (96.7%)	0.522 ^1^	57 (90.5%)	41 (95.3%)	98 (92.5%)	0.351 ^1^
	Yes	2 (4.8%)	2 (2.6%)	4 (3.3%)		6 (9.5%)	2 (4.7%)	8 (7.5%)	
Bleeding	No	38 (90.5%)	76 (97.4%)	114 (95.0%)	0.095 ^1^	53 (84.1%)	40 (93.0%)	93 (87.7%)	0.170 ^1^
	Yes	4 (9.5%)	2 (2.6%)	6 (5.0%)		10 (15.9%)	3 (7.0%)	13 (12.3%)	

^1^ Pearson’s Chi-squared test.

**Table 7:** Outcomes by NT-proBNP Levels and Cancer Status.

Outcome	Category	Oncological Group < 600 (N = 66)	Oncological Group > 600 (N = 40)	Oncological Group Total (N = 106)	*p*-Value	Non-Oncological Group < 600 (N = 92)	Non-Oncological Group > 600 (N = 28)	Non-Oncological Group Total (N = 120)	*p*-Value
Early Death	No	54 (81.8%)	33 (82.5%)	87 (82.1%)	0.9291	84 (91.3%)	20 (71.4%)	104 (86.7%)	0.0071
	Yes	12 (18.2%)	7 (17.5%)	19 (17.9%)		8 (8.7%)	8 (28.6%)	16 (13.3%)	
Late Death	No	40 (60.6%)	25 (62.5%)	65 (61.3%)	0.8461	88 (95.7%)	28 (100.0%)	116 (96.7%)	0.2621
	Yes	26 (39.4%)	15 (37.5%)	41 (38.7%)		4 (4.3%)	0 (0.0%)	4 (3.3%)	
Recurrence	No	59 (89.4%)	39 (97.5%)	98 (92.5%)	0.1261	88 (95.7%)	28 (100.0%)	116 (96.7%)	0.2621
	Yes	7 (10.6%)	1 (2.5%)	8 (7.5%)		4 (4.3%)	0 (0.0%)	4 (3.3%)	

**Table 8:** Factors Associated with Cancer Status in Acute PE: Multivariable Logistic Regression.

Predictor	Comparison/Unit	Estimate (β)	SE	z	*p*	OR (95% CI)
Intercept	—	−1.2374	0.8227	−1.5040	0.133	0.290 (0.0578–1.455)
NT-proBNP	>600 vs. <600	0.7317	0.3358	2.1788	0.029	2.079 (1.0763–4.014)
D-dimer	>5 mg/L vs. <5 mg/L	−1.0572	0.3141	−3.3656	<0.001	0.347 (0.1877–0.643)
Age	per 1 year	0.0243	0.0101	2.3998	0.016	1.025 (1.0045–1.045)
Sex	Male vs. Female	−0.4061	0.2869	−1.4154	0.157	0.666 (0.3797–1.169)
ECG changes	Yes vs. No	0.4003	0.2912	1.3750	0.169	1.492 (0.8434–2.641)
Hemodynamic status	stable vs. unstable	0.1958	0.3605	0.5431	0.587	1.216 (0.6000–2.465)
Pulmonary hypertension	Yes vs. No	−0.1956	0.3437	−0.5690	0.569	0.822 (0.4193–1.613)
Environment	Urban vs. Rural	0.0185	0.2938	0.0628	0.950	1.019 (0.5727–1.812)

Multivariable logistic regression estimating odds of oncological patients vs. non-oncological patients; predictors measured at presentation.

## Data Availability

The original contributions presented in this study are included in the article. Further inquiries can be directed to the corresponding authors.
